# Genomic Analysis of *Sleeping Beauty* Transposon Integration in Human Somatic Cells

**DOI:** 10.1371/journal.pone.0112712

**Published:** 2014-11-12

**Authors:** Giandomenico Turchiano, Maria Carmela Latella, Andreas Gogol-Döring, Claudia Cattoglio, Fulvio Mavilio, Zsuzsanna Izsvák, Zoltán Ivics, Alessandra Recchia

**Affiliations:** 1 Center for Regenerative Medicine, Department of Life Sciences, University of Modena and Reggio Emilia, Modena, Italy; 2 German Centre for Integrative Biodiversity Research (iDiv) Halle-Jena-Leipzig, Leipzig, Germany; 3 Institute of Computer Science, Martin Luther University Halle-Wittenberg, Halle, Germany; 4 Howard Hughes Medical Institute, Department of Molecular and Cell Biology, University of California, Berkeley, Berkeley, California, United States of America; 5 Genethon, Evry, France; 6 Max Delbruck Center for Molecular Medicine, Berlin, Germany; 7 Division of Medical Biotechnology, Paul Ehrlich Institute, Langen, Germany; Chang Gung University, Taiwan

## Abstract

The *Sleeping Beauty* (SB) transposon is a non-viral integrating vector system with proven efficacy for gene transfer and functional genomics. However, integration efficiency is negatively affected by the length of the transposon. To optimize the SB transposon machinery, the inverted repeats and the transposase gene underwent several modifications, resulting in the generation of the hyperactive SB100X transposase and of the high-capacity “sandwich” (SA) transposon. In this study, we report a side-by-side comparison of the SA and the widely used T2 arrangement of transposon vectors carrying increasing DNA cargoes, up to 18 kb. Clonal analysis of SA integrants in human epithelial cells and in immortalized keratinocytes demonstrates stability and integrity of the transposon independently from the cargo size and copy number-dependent expression of the cargo cassette. A genome-wide analysis of unambiguously mapped SA integrations in keratinocytes showed an almost random distribution, with an overrepresentation in repetitive elements (satellite, LINE and small RNAs) compared to a library representing insertions of the first-generation transposon vector and to gammaretroviral and lentiviral libraries. The SA transposon/SB100X integrating system therefore shows important features as a system for delivering large gene constructs for gene therapy applications.

## Introduction

The *Sleeping Beauty* (SB) transposon is a member of the Tc1/*mariner* transposon superfamily. Tc1/*mariner* elements are generally 1,300–2,400 bp in length and contain a single gene coding for the transposase that is flanked by terminal inverted repeats (IR). The IRs of SB host a pair of binding sites containing short, 15–20 bp direct repeats (DRs). Both the outer and the inner pairs of transposase-binding sites are required for transposition. The SB transposase binds the IRs in a sequence-specific manner, and mediates precise cut-and-paste transposition in a wide variety of vertebrate cells including human cells [1]–[3]. For this reason, the SB-based integration system is a valuable tool for functional genomics in several model organisms and represents a promising vector for human gene therapy [4], [5]. However, a major bottleneck of any transposon-based application is the low transposition efficiency. Therefore, considerable effort was dedicated to improve the SB integration machinery by modifying its IRs and systematically mutating the transposase gene. In 2002, Cui et al. carefully explored the structure and functions of the IRs. They modified the outer and inner DR sites of both IRs and the spacer sequence between the DRs generating a new version of transposon IR, called T2, with fourfold increased transposition efficiency [6]. However, the transpositional activity of this system (and that of the first-generation transposon [7]) is negatively affected by the size of transposon, resulting in an exponential drop for every kb introduced between the two IR.

In 2004, Zayed et al. constructed the “sandwich” (SA) version of the transposon vector [8]. The SA IR consists of two complete transposon elements in a head to head orientation, flanking a DNA expression cassette, thereby forming a sandwich-like arrangement. Mutation of the 5′ terminal CA nucleotides of the right IR abolishes cleavage at the innermost transposon ends; therefore, only the four terminal DRs represent the catalytic substrate for the “cut and paste” transposition. The SA transposon showed a 3.7-fold enhanced activity over first generation transposon to integrate ∼7.5 kb-DNA sequence upon SB10 transposase delivery. Five years later, a transposase 100-fold more active than SB10, named SB100X, was developed by a high-throughput, PCR-based DNA shuffling strategy [1]. The improved integration efficiency associated with SB transposition opened new avenues for its application. The hyperactive SB100X transposase was employed to obtain highly efficient germline transgenesis in pigs [9], [10] rabbits [11] and rodents [12], [13], stable transfer of therapeutic genes in clinical relevant cells [1], [14]–[18], and reprogramming of mouse embryonic and human foreskin fibroblasts into iPS cells [19].

In this study, we investigated the integration efficiency of large expression cassettes mediated by the optimized SB elements: the SA transposon and the SB100X transposase. We report a side-by-side comparison between the SA and the T2 transposons carrying DNA cargo of increasing length. We performed a deep molecular characterization of SA-mediated integrants in epithelial cell lines and in primary immortalized keratinocytes stressing the SB system with cargos up to 18 kb. These data provide evidence for stability of SB-mediated integration and the reproducibility of the cut-and-paste mechanism even with large transposons embedded between two double IRs. Moreover, clonal analysis reveals a linear correlation between transposon copies harboured into the genomic DNA and their expression, an important characteristic for gene therapy application. Finally, high-resolution, genome-wide mapping of SA integrations in human keratinocytes revealed a close-to-random integration pattern with respect to genes and chromosomes, highlighting a relative low risk of genotoxicity as previously reported for SB transposition in cell lines [20]–[23]. Interestingly, the high-throughput analysis of SA integration sites showed an overrepresentation of integration events into repetitive elements (RE) of the human genome, in particular satellite, small RNA and LINE elements.

## Materials and Methods

### Cell culture

HeLa cells were cultured using DMEM medium (Lonza) added with 10% Fetal Bovine Serum (FBS), 1% L-Glutamine (L-Gln) and 1% Penicillin-Streptomycin (Pen/Strep). For each experiment, an aliquot of cryo-preserved HeLa cells was thawed and plated on 8 cm dishes. Upon reaching 80–90% of confluency, cells were re-plated on 6-wells culture plates at a concentration of 2×10^5^ cells/well. After 24 h, cultures in each well were at 70–80% confluency, ready to be transfected.

Mouse NIH3T3 fibroblast cell line was maintained in Dulbecco's Modified Eagle's medium (Euroclone), supplemented with 10% bovine serum.

We have used SV40 immortalized keratinocytes derived from a patient affected by generalized atrophic benign epidermolysis bullosa (GABEB) produced by Borradori et al. [24] and kindly provided by J.W. Bauer. GABEB cells were cultivated in EpiLife medium supplemented with human keratinocyte growth supplement (HKGS) (Invitrogen, US). EpiLife is a serum-free keratinocyte culture medium with a low calcium (0.06 mM) concentration supplemented with HKGS which results in a final concentration of 0.2% (v/v) BPE, 5 lg/mL bovine insulin, 0.18 *lg/mL* hydrocortisone, 5 lg/mL bovine transferrin and 0.2 ng/mL human EGF. Upon reaching 80–90% of confluency, cells were re-plated on 6-wells culture plates at a concentration of 2.3×10^5^ cells/well. After 24 h, cultures in each well were at 70–80% confluency, ready to be transfected.

### Plasmid constructs

The plasmid carrying the T2 IRs including a Venus reporter gene driven by the chicken β actin promoter fused to CMV early enhancer element (CAGGS) and the construct coding for the SB100X were described in Mates et al. [1]; the SA transposon IRs were described in Zayed et al. [8]. The CAGGS Venus expression cassette was *Dra III* excised from pT2 3.2 and introduced into *EcoRV* digested pSA to obtain pSA 5.7. pT2 3.2 and pD28 [25] were digested with *XbaI* to clone a non coding DNA of 2.7 kb from pD28 into the transposon.

Two fragments of the first intron of the HPRT gene were PCR amplified and cloned into the pCR 2.1 (TOPO cloning kit, Invitrogen) plasmid. The pT2 10 plasmid was cloned ligating the pT2 CAGGS Venus *SpeI* with *NheI* fragment of the amplified HPRT intron 1. The pT2 14 plasmid derives from pT2 10 digested with *ClaI* ligated to the *NotI* fragment of the amplified HPRT intron 1. Finally, pT2 18 was obtained by ligating a third sequence amplified from the HPRT intron 1 with pT2 14 through *EcoRI* restricted ends. The pSA 5.7 plasmid was digested with *NheI* and ligated to the *NheI* non coding fragment of the HPRT gene to obtain the pSA 9.7. Then the pSA 9.7 was digested with *PmeI* enzyme and ligated with a *PvuII* fragment of the HPRT intron 1 to obtain the pSA 14. To enlarge the pSA14, a sequence amplified from the intron 3 of the Lamb3 gene was introduced by *EcorV* compatible ends to obtain pSA 18.

### Transfection-based transposition and calculation of transposition efficiency

HeLa and GABEB cells were both transfected with FugeneHD transfection reagent (Roche). For each sample 2 µg of DNA were added to 100 µl of either DMEM (for HeLa) or EpiLife (for GABEB). The media used for this transfection reaction mix were not added with FBS, L-Gln or Pen/Strep.

The transposon/transposase amounts of plasmid DNA were calculated to respect the stoichiometric ratio of 1∶1 or, for transposon >10 kb, 2∶1, in a total quantity of 2 µg. 2 µg of transposon-only plasmid were used for non-transposed control.

Each transfection reaction mix was complexed with 6 µl of FugeneHD (10 µl with SA and T2 18 in GABEB cells) and subsequently mixed by pulse-vortexing for a few seconds. The mixes were thereafter left at room temperature for 10′ in order to allow the formation of lipoplexes. After the 10′ had expired, each mix was added drop-by-drop to a cell culture sample, which was subsequently incubated at 37°C.

HeLa cells were transfected with Calcium Phosphate method using 15 µg of 14- or 18 kb transposons mixed with the plasmid carrying the transposase expression cassette.

The percentage of Venus^+^ cells was determined 2 and 20–30 days post-transfection via flow cytometry and the transposition efficiency was calculated as: Venus^+^ cells at 20–30 days post transfection/Venus^+^ cells at Day 2×100. Cells that were only transfected with the transposon plasmid represented the control for background integration events.

Transposed clones were analysed via flow cytometry to determine the presence of doublets and the Venus mean fluorescence intensity (MFI).

### Isolation of single cell clones

GABEB cells were limiting diluted to obtain a concentration of 0.5 cell/well, plated onto lethally irradiated NIH3T3 cells and cultured in keratinocyte growth medium, a DMEM and Ham's F12 media mixture (2∶1) containing FCS (10%), penicillin-streptomycin (1%), glutamine (2%), insulin (5 µg/ml), adenine (0.18 mM), hydrocortisone (0.4 µg/ml), cholera toxin (0.1 nM), and triiodothyronine (2 nM). After 1 week, the medium was replaced by EpiLife medium supplemented with HKGS. After 2 weeks GABEB cells were trypsinised at subconfluence and re-plated without the NIH 3T3 feeder-layer in EpiLife HKGS medium.

HeLa cells were seeded to obtain a concentration of 0.3 cells/well in a 96 well plate in DMEM medium complemented with 10% FBS.

### Southern blot analysis

Ten µg of genomic DNA, extracted from 1−5×10^6^ cells by a QIAmp DNA Mini kit (Qiagen), were digested overnight with *NheI* (SA 9.7-derived clones) and *AflII* (T2 10-derived clones) to verify the copy number of the transposed cassette, or with *NcoI* (SA 9.7-derived clones) and *MfeI* plus *NdeI* (T2 10-derived clones) to verify the integrity of the transposed cassette. Digested gDNA was run on a 0,8% agarose gel, transferred to a nylon membrane (Duralon, Stratagene) by Southern capillary transfer and probed with 2×10^7^ cpm ^32^P-labeled Venus probe according to standard techniques [26].

### PCR screening for episomal SB vectors

About 100 ng of template gDNA were used in a PCR reaction. Primers capable to amplify the Amp resistance gene or the SB100X transposase (**Table S1**) were used to detect genomic integrations of SA 9.7 backbone and SB100X, respectively. PCR conditions were as follows: 30′′ at 94°C, 30′′ at 58°C and 30′′ at 72°C for 30 cycles.

### LM-PCR and bioinformatic analysis

Integration sites were amplified by Linker Mediated PCR (LM-PCR), as described [27]. Briefly, genomic DNA was extracted from 0.5−5×10^6^ transposed cells and digested with *Mse*I and *XhoI* enzyme to prevent amplification from internal mutated IR fragments. An *Mse*I double-stranded linker was then ligated and LM-PCR performed with nested primers specific for the linker and SA IR/DR (**Table S1**).

LM-PCR derived amplicons were run on a Roche/454 GS FLX using titanium chemistries by GATC Biotech AG Next Gen Lab. A valid integration contained: the TAGpSAIR nested primer and the entire SA IR/DR sequence up to a TA dinucleotide.

#### Alignment pipeline

31,603 sequencing reads were tested for the presence of the SA IR sequence and TA dinucleotide. The SA IR and any primer sequences were trimmed, and the remaining reads starting with TA dinucleotides were mapped to the human genome (hg19) using NCBI BLAST (blastn with default parameters). We kept only reads which were mapped to a single genomic site with at least 90% sequence identity and an E-value of at most 0.05. Only reads which could be mapped from their 5′ end onwards were considered for further analysis. Redundant reads mapping to identical genomic positions were collapsed. This way we got 2019 unique SA integration sites.

For the statistical analysis we generated 10,000 control sites in-silico taking into account the bias introduced by LM-PCR techniques. We first generated artificial reads starting with TA dinucleotide of the human genome in a way that the control sequences had both the length and the frequency of *MseI* restriction sites (TTAA) as observed in real sequencing reads. The artificial reads were then processed by the same mapping criteria used for the SA sites.

#### RM blast analysis

Analyses of repetitive element were performed with RepeatMasker Blast (http://repeatmasker.org) [28]. To achieve reliable and comparable results we processed the raw sequences trimming out the primer sequences used in LM-PCR, the IR/LTR/linker specific sequences following the primers. Resulting reads were further trimmed till the 40^th^ nucleotide discarding every sequence with less than 40 nucleotides. Finally, we collapsed the reads that were either identical or with one mismatch. A two-sample test for proportions was used for pairwise comparison of the RE within the different datasets.

For statistical analysis we created control sets as follows. We first randomly sampled 1 Million sequences 49 bp in length from the human reference genome (hg19). Then we discarded all sequences not starting with TA. The resulting set of 65,826 TA-weighted sequences was used as a background for T*neo* SB integrations. For a second random control set we first randomly sampled 10 Million sequences of length 120 bp from the genome. Then we discarded all sequences not starting with TA, or either not containing the *MseI* restriction motif TTAA or having a TTAA within the first 39 bp of the sequence. After removing the part of the sequences following the first occurrence of TTAA, we received 292,917 sequences of lengths between 40 bp and 120 bp, which were weighted for TA and *MseI* and could be used as a background for SA integrations. We passed the generated sequences through the same filtering/trimming pipeline as the actual integration reads.

A third random control set of 45,235 genomic sequences weighted for *MseI* was adapted from Cattoglio et al. [29] and used as a background for MLV and HIV integrations.

### Bidirectional PCR mapping on GABEB clones

Transposon integrations in GABEB clones were amplified by LM-PCR as described. PCR products were shotgun-cloned (TOPO TA cloning kit, Invitrogen) and then sequenced. Sequences between the TA and the linker primers were mapped onto the human genome by the BLAT genome browser (UCSC Human Genome hg19). Sequences featuring a unique best hit with ≥90% identity to the human genome were considered genuine integration sites. To confirm the genuine integration in both directions we design primers on the genomic region hit and performed a direct PCR in conjunction with the pSAIR specific primer for the SA IR sequence (**Table S1**). The derived amplicons were loaded on agorose gel and checked for the expected length.

## Results

### Efficiency of T2 and SA transposons

The sandwich (SA) transposon vector has superior ability to transpose >10 kb transgenes with respect to the first-generation transposon when SB10 transposase was provided [8]. Nevertheless, the T2 transposon, resulting from site-specific mutations in the IR sequences and insertion of double TA flanking each IR, has been demonstrated to have a four-fold enhanced activity over the first-generation transposon construct [6]. A side-by-side comparison of SA and T2 transposon was needed to address the transposition efficiency of increasing DNA cargoes and to verify their molecular behaviours once integrated into the human genome.

**Figure 1:**
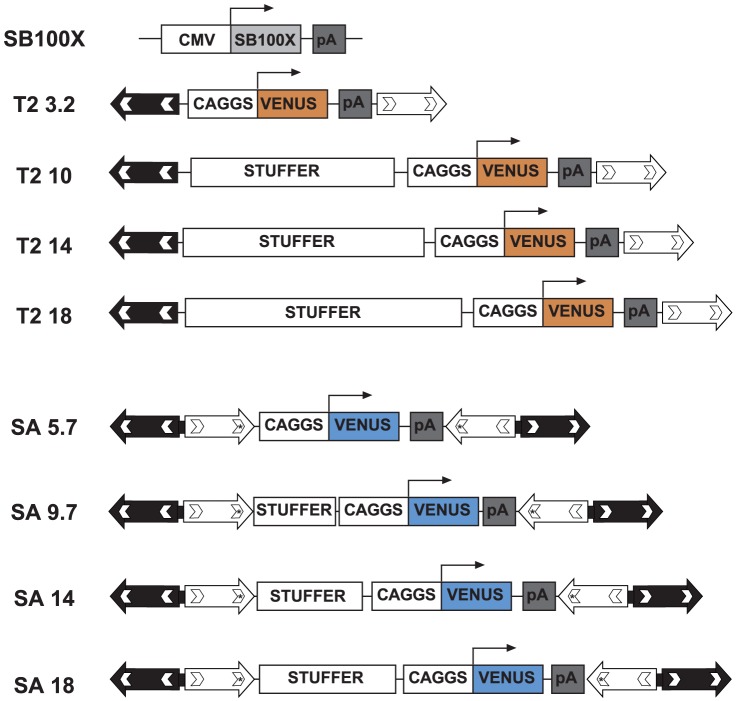
Transposon fleet. Schematic representation of the generated plasmids. SB100X carries the Hyperactive *Sleeping Beauty* transposase coding sequence placed under the control of the CMV promoter and followed by an SV40 poly-Adenylation (pA) signal. The transposons T2 and SA possess the expression cassette consisting of the CAGGS promoter, VENUS reporter gene and SV40 pA signal. The stuffer DNA represented has variable increasing size. The arrows represent the IR/DR ends recognised by the transposase. SA constructs are characterized by the presence of two complete IR/DR at each ends (white and black arrows) and the asterisks underline the IR mutated site not recognized as a catalytic substrate by the transposase. Numbers following T2 or SA abbreviation indicate the size in kilobases of the transposed cassette.

We generated SA- and T2-based plasmids (SA 5.7 and T2 3.2 **Figure 1**) keeping the Venus reporter gene as standard expression cassette. Increasing sizes of a non-coding human stuffer DNA (4-, 8.3- and 12.3 kb in the SA plasmid; 6.8-, 10.8- and 14.8 kb in the T2 plasmid) were introduced between the two IR/DR to produce transposons of comparable length. For the sake of simplicity, we named these plasmids with the transposon construct type and the size of the transposable cassette expressed in kilobases (**Figure 1**).

Transposition experiments were performed in HeLa cells and in immortalized primary keratinocytes derived from patients affected by Generalized Atrophic Benign Epidermolysis Bullosa (GABEB), an inherited skin adhesion defect. All the experiments aimed at the identification of the integration efficiency of the IR-flanked transgene were measured by long-term Venus fluorescence in the absence of selective pressure. We co-transfected the SB100X transposase-expressing plasmid together with transposon plasmids in two different molar ratios (1∶1 or 1∶2) depending on the transposon length. Larger cargos required more transposon DNA to reach good transfection efficiency.

At least three independent experiments for each cell type and transposon were performed in order to reduce variability due to the transfection procedure. Mock-transfected HeLa and GABEB cells, and cells transfected with the T2 or SA Venus constructs alone were used as controls (in the absence of transposase, no transposition event should occur and residual reporter gene expression after long periods would only be attributable to noise or to rare random plasmid integration events). Transgene expression all along the culture period (up to 31 days) was measured via flow cytometry to follow the trend of the signal that persists in presence of SB100X and drops without the transposase (**Figure S1)**.

The transposition efficiency was normalized by transfection efficiency (numbers of cells that received the plasmids after transfection) and calculated as the ratios between the percentage of Venus^+^ cells at the endpoint (20–31 days) and the percentage of transfected cells 2–3 days after DNA delivery to the cells. The endpoint of each experiment is achieved when the percentage of Venus^+^ cells in the sample transfected with the transposon alone stabilized to less than ∼0.5%.

**Figure 2:**
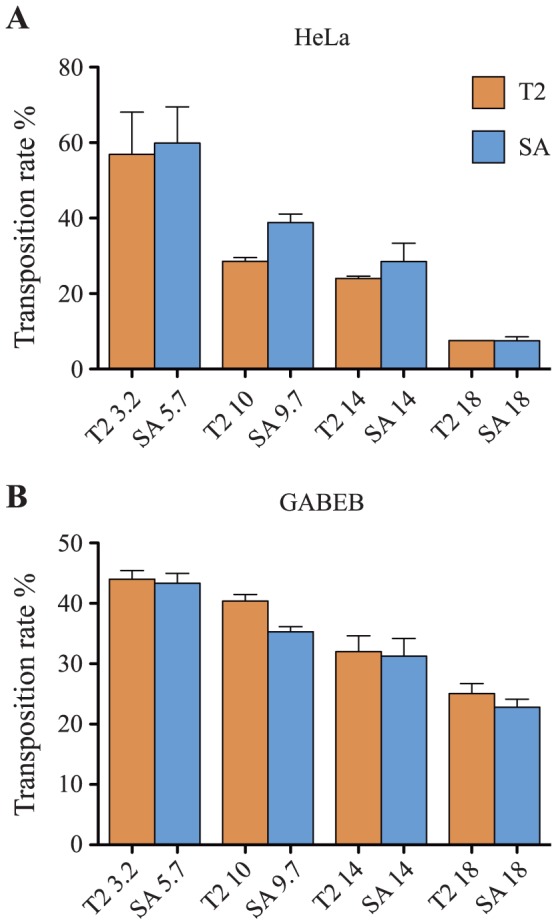
Transposition efficiency. HeLa (A) and GABEB (B) cells were co-transfected with the T2 and SA transposons- and transposase-carrying plasmids. The transposition rate, on the Y axis, is derived by the ratio between the percentage of Venus^+^ cells at about 20 and 2 days post transfection. Data are representative of three independent experiments (mean ± SEM; *n* = 3).

**Figure 2A and 2B** show the transposition rate obtained in HeLa and GABEB cells. As previously reported [1], [8], the transposition efficiency was inversely proportional to the transposon size. In HeLa cells, the transposition efficiency dropped 7.8 fold (from 58.5% to 7.5%) when increasing the cargo payload from 3.2 kb to 18 kb, independently of the transposon structure (T2 or SA). Interestingly, this size-dependent effect was less pronounced in GABEB cells. In this cell type the decrease was of 1.8 fold (from 44% to 24%) for T2 and SA and the transposition rate for 18 kb transposons remained approximately 24% compared to the 7.5% in HeLa cells.

### Clonal molecular analysis

Although we performed a molecular characterization of almost all T2 and SA vectors in HeLa or GABEB cells (**Table S2**.), we focused our genomic analysis on a relatively large T2 and SA transposons cassette (10 kb) and on GABEB keratinocytes. Bulk populations of transposed cells were sorted for Venus expression 20–35 days post transfection and cloned by limiting dilution. Genomic DNA extracted from each clone was first investigated by PCR for the presence of the transposon backbone and SB100X expressing plasmid. Notably, we scored 14.8% of clones (8 out of 54) positive for the Ampicillin sequence present within the transposon backbone about 60 days post transfection, while few (2 out 54) of the analysed clones were positive for the SB100X sequence (**Table S2**).

**Figure 3:**
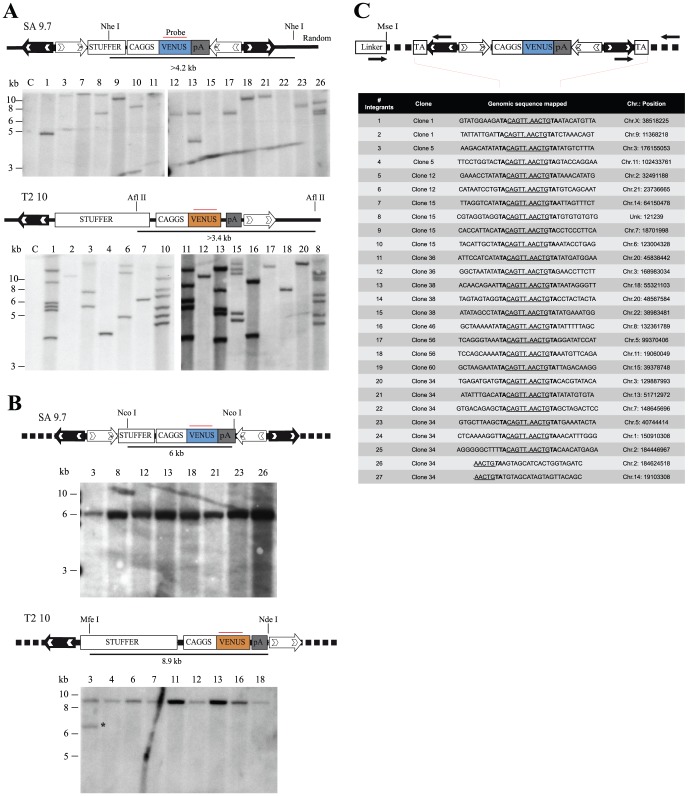
Molecular characterization of the *Sleeping Beauty*-mediated integration events in GABEB cell clones. (A) Southern blot analysis of genomic DNA from GABEB cell clones digested with *NheI* (SA clones) or *AflII* (T2 clones), single cutter in the transposon cassette, and hybridized to a Venus probe. A single band higher than 4.2 kb (SA clones) and 3.4 kb (T2 clones) indicates integration of one copy of the transposon into the genome. Multiple Venus-specific bands correspond to repeated integration events. (B) Southern Blot analysis of genomic DNA from 8 (SA) and 9 (T2) clones digested with *NcoI* (SA clones) or *MfeI* and *NdeI* (T2 clones). The expected Venus-specific band corresponding to 6 kb for SA and 8.9 kb for T2 transposon indicates the correct integration of the transposons into the genome. C, mock-transfected cells; red bars, Venus-specific probe. Clone showing rearrangement of the transposon cassette is highlighted by black asterisk. (**C**) Bi-directional mapping of the junctions between transposon and genomic DNA. The table summarizes 27 integrations belonging to 10 single clones. For each integrant, the underlined sequence represents a portion of the transposon IRs, left (CAGTT) and right (AACTG) separated by dots; TA dinucleotide (in bold) is the target site correctly duplicated after transposition. Hit chromosomes and positions are reported. UnK, unknown region of the human genome based on UCSC hg19 assembly.

We next performed Southern blotting on the genomic DNA of 16 clones for each transposon type to determine the transgene copies harboured in the genome and their integrity. To this end, we digested the genomic DNA with *AflII* (T2 clones) or *NheI* (SA clones) that release fragments longer than 3.4 and 4.2 kb. Hybridization with a Venus-specific probe showed that most of the SA treated samples (13 out of 16) carry a single integrated transposon, only 1 clone (#26) had 3 copies, and 2 out of 16 clones contained 2 copies (#8, #13) resulting in an average copy number of 1.3. Surprisingly, 16 GABEB clones obtained with T2, harbour 1 to 7 copies with an average of 3 integrated transposons per clone **(** **Figure 3A** **). In general we observed that the mean copy number is more affected by the transfection efficiency (Table S2) respect to the size and type of transposons.**

Further restriction analysis performed with *MfeI* and *NdeI* on 9 T2 clones and with *NcoI* on 8 SA clones showed that all clones harbour the full-length transposon cassette (**Figure 3B**). Among the 21 integrated transposons in the 9 T2 clones, only one, belonging to clone #3, is shorter than expected. None of the 13 integrated transposons in the 8 SA clones was rearranged.

To unequivocally prove that all the integration events mediated by SA transposition resulted from a genuine “cut and paste” mechanism, we mapped the insertion site at both transposon ends using an adapted version of Linker-Mediated PCR (LM-PCR) [27]. Ten Venus-expressing GABEB clones, derived from transposition of the SA 5.7 plasmid, were examined. Six integrants (#1, 4, 7, 13, 14, 16) belonging to 5 clones were bi-directionally mapped by LM-PCR. Additional 21 integrants were revealed by LM-PCR and confirmed by specific PCR on the genomic region flanking the opposite IR (**Figure 3C**). Importantly, almost all the integration events occurred without genomic rearrangements, deletions or insertions, in the target sites. Only 2 out of 27 integrations (#26 and #27 belonging to clone 34) could not bi-directionally confirmed.

**Figure 4:**
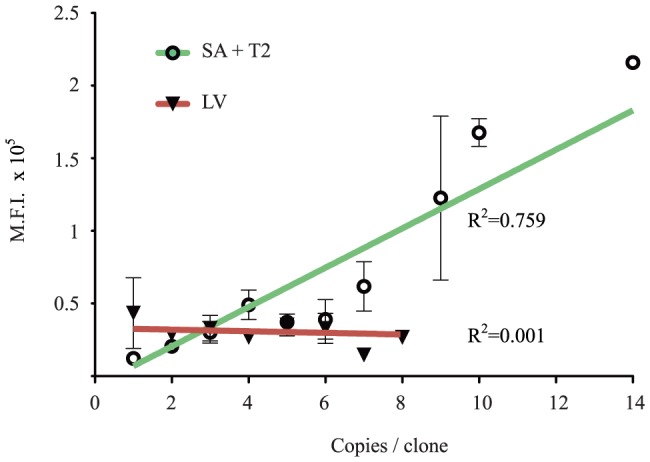
Correlation between copy number and expression of the integrated cassette. Mean fluorescence intensity (M.F.I.) of 62 GABEB clones positive for Venus expressing SB transposons are represented as circles; triangles indicate the M.F.I. of 70 GFP^+^ HaCaT clones transduced with a lentiviral vector (LV). Standard deviation bars are present for those clones carrying the same copy number. R^2^ coefficients of determination were extracted from the linear regression plot, green line for SA and T2 transposons and red line for LV.

Finally, we correlated the expression level of the reporter gene with the copy number of the transposon. The positional effect variegation primarily observed with retroviral and lentiviral vectors [30] could lead to the silencing of the therapeutic gene delivered by the vector. We asked weather the SB integrations would be affected by this phenomenon. We correlated the expression of Venus protein, measured by Mean Fluorescence Intensity (M.F.I.), with copy number of either the SA and T2 transposon, as determined by Southern blot or q-PCR analyses of 62 GABEB clones. For comparison, we analysed the M.F.I of a GFP reporter gene, driven by the human Keratin 14 promoter, in 70 HaCaT clones isolated upon LV transduction. A linear correlation curve was traced to retrieve the R^2^ coefficient of determination. Transposon samples show an R^2^ = 0.759 with a statistically defined correlation between two variables (P_N_ = 0.6). LV samples display an R^2^ = 0.001 with a null defined correlation (**Figure 4**). Independent analysis of transposed clones obtained in different cells (HaCaT and GABEB) and carrying a reporter gene driven by PGK or CAGGS promoter showed comparable results indicating common directly correlation between MFI and copy number (data not shown). We conclude that SB integrants tend to express their cargo faithfully, and multi-copy integrants express in a copy-number dependent manner, consistent with earlier observations [31].

### Integration pattern analysis

**Figure 5:**
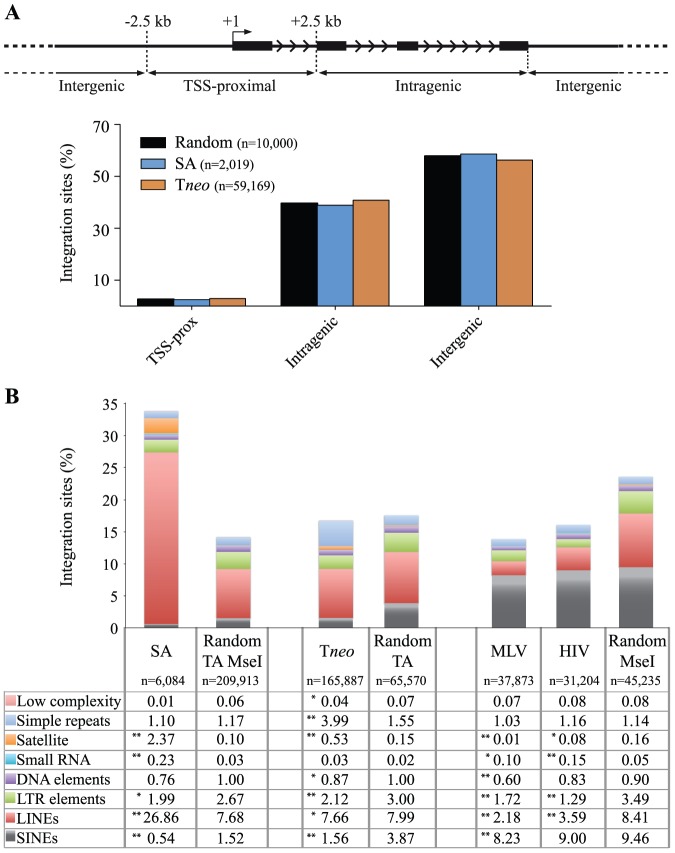
Integration pattern analysis. (A) Integration sites were annotated as “TSS-proximal” when occurring within a distance of ±2.5 kb from the gene's TSS, as “Intragenic” when occurring in a gene body and as “Intergenic” in all other cases. Black bars represent exons of a schematic gene, arrowhead indicates the direction of transcription. Distribution of SA, T*neo* and random integration sites in the genome is plotted accordingly to defined annotations. (B) Distribution of Repetitive Elements in SB SA and T*neo* libraries, in MLV and HIV libraries. Relative weighted random libraries were reported: TA and *MseI*-weighted for SA, TA-weighted for T*neo* and *MseI*-weighted for MLV and HIV libraries. **p≤10^−3^, *p≤10^−2^.

In the last few years, several papers described the integration profile of the SB, *piggyBac* (PB), and Tol2 transposons [20]–[23], [32]–[36]. Here we report the integration profile and preference of the sandwich compared with the first-generation SB transposon [20] in human epithelial cells. To generate a library of SA integration events, we transfected 20 million GABEB cells with SA transposon- and SB100X-carrying plasmids. The 20% of Venus-positive cells were sorted three days after transfection to enrich the population expressing the reporter gene. A 90%-pure sorted population was kept in culture for 3 weeks to dilute the un-integrated SA vector reaching a stable 78% Venus^+^ bulk population. We used LM-PCR and pyrosequencing to generate 6,084 non-redundant SA-linked genomic sequences in human immortalized GABEB keratinocytes. The Blast alignment retrieved 2,019 unambiguously mapped integration sites. As a control, 10,000 random unique sequences were generated in silico balancing the biases introduced by the LM-PCR (amplicon lenght and *MseI* proximity) and the availability of the TA dinucleotides in the genome. In the analysis we also annotated a large dataset (59,169 hits) generated in HeLa cells transposed with the first-generation T*neo* transposon and selected for 2 weeks with neomycin [20]. The integration sites and control sites were annotated as transcriptional start site (TSS)-proximal when mapping in the ±2.5 kb window around a TSS, intragenic when mapping within a transcription unit, and intergenic in all other cases. Among SA integrations, 58.6% were in an intergenic position, 38.9% were within the transcribed portion of at least 1 gene, and 2.5% was within a 5 kb window encompassing the TSS (**Figure 5A**; the complete list of sequences is available in GenBank database with the accession number SRP047118). In general, the distribution of the SB integrants in both datasets is fairly random and resembles the composition of the human genome showing no statistical differences compared to their relative controls, i.e. all p-values (both two-sample tests for proportions and Fisher's Exact Tests) were >10^−2^.

We then analysed the frequency of human repetitive elements in the transposon libraries, SA and T*neo* [20], and their relative weighted controls availing of the RM Blast browser [28]. For comparison we also analysed two viral-derived integration datasets (MLV and HIV) generated in human CD34^+^ multipotent hematopoietic progenitor cells (HPCs) [29] and their control library weighted for *MseI* restriction site distribution. The raw data generated by deep sequencing of the LM-PCR (applied to SA, MLV and HIV treated cells) and LAM-PCR (applied in [20]) products were filtered and trimmed in order to rescue the genuine integration events (see materials and methods). After filtering and trimming we retrieved 6,084 and 165,887 unique sequences in SA and first-generation vector libraries, respectively, and 37,873 and 31,204 unique sequences from MLV and HIV datasets, respectively. We generated large control datasets taking into account the bias introduced by the respective technique. In particular from the hg19 genome database we retrieved 45,235 control reads weighted for *MseI*, 65,570 sequences weighted for the presence of TA dinucleotide hit by the SB transposons, and 209,913 sequences *MseI-* and TA-double weighted.

The RM Blast analysis revealed an overrepresentation of REs in the SA integrations (34%) with respect to the TA and *MseI*-weighted control (14%) and to all the other datasets analysed (**Figure 5B**). In particular, Satellite, small RNA and LINE elements were enriched in the SA library (24-, 7.6- and 3.5-fold increase over the background, respectively) whereas in the first-generation vector library only a slight increase in the satellite and simple repeats elements was measurable (3.5- and 2.6-fold over the background, respectively); comparable LINE frequency was detected.

Besides the higher frequency in the satellite elements, the two SB transposon datasets share a slight under-representation of SINE, LTR and DNA transposable elements in comparison with their random control libraries. We introduced MLV and HIV libraries to compare the frequency of integration into RE generated by a retroviral integrase-mediated integration mechanism. The RM Blast analysis pointed out that viral vectors disfavour integration in RE (14–16% vs 24%), and, in particular, satellite, LTR and LINE elements are underrepresented. These data clearly confirm a difference in the integration site selection between viral vectors and SB transposons and identify new signatures in the SA integrome that should be taken into consideration when using them as tools for genetic manipulation.

## Discussion

The SB transposon IRs were mutated to improve their capacity to be mobilized and, to date, there is not a direct comparison that define genetic characteristics of the T2 and SA IRs [8]. In this study, HeLa cells and GABEB keratinocytes [24] were transfected with a panel of T2 or SA transposons carrying size-increasing Venus expression cassette in combination with SB100X plasmid (Figure 1). Transfection rate was higher in HeLa than in GABEB cells (**Figure S1**) and the transposition efficiency was inversely proportional to the transposon size (**Figure 2**). Interestingly, HeLa cells were severely affected by the transposon size compared to primary immortalized cells. These results suggest that the transposase activity could be favoured by some cellular factor differentially expressed in GABEB and HeLa cells. Nonetheless, T2 and SA constructs carrying cargos of comparable size showed similar transposition efficiency in both cell lines. From these data we can conclude that the T2 IR construct is interchangeable with the SA construct with some advantages: T2 has shorter IRs thereby it could accept a larger cargo cassette.

Transposed GABEB and HeLa populations were subjected to limiting dilution to obtain a single cell derived expansion. The derived clones were employed to characterize several molecular parameters: transposon-independent insertion, copy number, genomic stability, faithful transposition activity, correlation between copy number and expression of the integrated cassette. The SB100X sequence was retrieved in 6 out of 211 analysed clones while almost 14% of the clones (30 clones) were found positive for the transposon backbone sequences (**Table S2**). We hypothesize that the plasmid backbone carrying the transposon could have some advantages to remain episomal or to integrate in the genome. The transposon excision step from the plasmid leaves the backbone with a double strand break that induce recruitment of the endogenous repair machinery and integration into the cell genome. We also analysed the copy number of the clones. **Figure 3A** shows an average of 1.3 SA copies/clone while T2 copy number spans from 1 to 7 transposons with an average of 3 copies. This difference mostly depends on the transfection efficiency as confirmed by the analysis of the other transposed cell populations generated in this study. Therefore, it is possible to fine tune this parameter by adjusting the ratios of the two SB components used for transfection or, as previously reported, bypass the transfection procedure through the viral delivery of transposase and transposon by adenoviral vector [37], integration defective lentiviral vector [38], [39], retroviral particle [40] and adeno-associated vectors [41].

We were able to associate copy number of the transposon with the expression level of the Venus fluorescence gene. Mean Fluorescence Intensity does follow a direct proportion with the copies harboured (**Figure 4**). In contrast, expression of the reporter gene in lentiviral-mediated integrants does not correlate with copy number and is more subjected to the activity of surrounding genomic sequences [42], [43].

Next, the integrated transposons in these clones were also analysed for their integrity via Southern Blot. Retroviral and lentiviral vectors can rearrange during the reverse transcription step resulting in partially-deleted integrated proviruses, a frequent occurrence in transgene hosting repetitive sequences [44], [45]. The SB mediated integration, by contrast, does not require reverse transcription and thus is expected to preserve the integrity of the transgene. Ninety-eight percent of the integrants, resulting from T2 and SA transposition, have a correct size (**Figure 3** **B**).

The sandwich transposon has a doubled IR/DR structure at both ends with 8 transposase binding sites in total. In principle, every transposase unit, bound to one DR site, could interact with the others to create different chiasm geometries (also described in [6]); some of these conformations could modify the integration activity resulting in chromosomal aberrations. To investigate the fidelity of the transposition process 10 GABEB clones were mapped bi-directionally by LM-PCR and transposon-genome junction was amplified by site-specific PCR. Twenty-five integrations, out of 27 (92.6%), were validated for a canonical transposition event with the TA target site duplication signature at both ends (**Figure 3C**). Two integrations mapped by LM-PCR were not confirmed in the opposite transposon end suggesting rearrangements probably caused by the repair mechanism occurred in the transposition break.

LM-PCR was also employed to derive a high-definition map of SA/SB100X integration sites in the genome of a transposed GABEB bulk population. This analysis is commonly applied to integrating vectors (i.e. retroviral and lentiviral vectors) because it allows to evaluate genotoxicity [46], [47] and to understand molecular mechanism driving the integration towards specific regions of the genome [29], [48]–[50]. The technique returned 2,019 SA unambiguously mappable integration sites randomly distributed throughout the human genome, in accordance with previously published data on first-generation transposon [20], [23] (**Figure 5A**). For gene therapy purposes, the SB system results in a safer integration profile compared to other integrating vector such as Tol2, PB transposon and retroviral vectors [20]–[23], [32], [33], which favor TSS-proximal regions or gene body sequences.

Although the integration site distribution in relation to genes was found close to random, the RM Blast analysis shows a significant bias distribution of SA integrations in repetitive elements (RE), particularly in satellite, LINE and small RNA genes. It could be that these genomic regions are favourable for integration due to their base composition (TA-richness) or there might be molecular mechanisms that actively recruit the transposon/transposase complex at specific RE sites [51]–[55].

Curiously, the frequency of RE elements in the first-generation transposon library and its weighted control were comparable. Differently from the SA (obtained in 80% Venus expressing immortalized keratinocytes without selective pressure), the first-generation transposon library derives from transposed HeLa cells [56] selected for two weeks by antibiotic resistance. This culture condition could negatively select those integrations landing into poorly expressed genomic loci or into heterochromatin regions [35]. Nevertheless, the first-generation transposon integrations were slightly increased into satellite regions and SINE, whereas LTR and DNA elements were underrepresented compared to the background.

These data identify some common features in SB datasets. Conversely, MLV and HIV-derived viral vectors disfavour integration in RE (satellite, LTR and LINE accordingly also to [57]) suggesting an active role of viral integrase in the selection of integration sites that could better support the expression, replication and survival of the viral progeny. The genomic features newly identified in the SA integrome raise an interesting matter that needs to be deeply investigated for future application.

## Supporting Information

Figure S1 **Transposition trend.** Expression of Venus fluorescence protein was detected by cytofluorimetric analyses at different time points in HeLa cells (A) and in GABEB cells (B). The days post transfection (p.t.) are plotted on the X axis, while the percentage of Venus+ cells are represented on the Y axis. The maximum expression from a transfected reporter gene was achieved 2 days p.t (black vertical dotted line). The plot shows the samples co-transfected with SB100X and transposon plasmids: T2 3.2 or the SA 5.7 (blue), T2 10 or SA 9.7 (green), T2 14 or SA 14 (purple), and the 18 kb transposons (orange). SA constructs represented with dashed lines, T2 with continuous lines. In gold are represented the negative controls transfected with T2 3.2 or SA 5.7 alone, without the SB100X plasmid.
(EPS)

Table S1 **List of primers used for plasmid episomial amplification, LM-PCR, and site-specific amplification of the SA-genome junctions.**
(DOC)

Table S2 **Transposed clones were analysed to show the following parameters: number of retrieved Venus^+^ clones for each bulk; percentage of Venus^+^ cells in bulk populations 48 hours p.t.; percentage of stable Venus expressing cells in bulk populations; percentage of clones positive for the Ampicillin or SB100X sequence carried by transfected plasmids; mean copy number retrieved by Southern blot analysis; recombinant events detected in transposed clones by Southern blot analysis.**
(DOC)

## References

[1] Mates L, Chuah M, Belay E, Jerchow B, Manoj N, et al (2009) Molecular evolution of a novel hyperactive Sleeping Beauty transposase enables robust stable gene transfer in vertebrates. Nature genetics 41: 753–761. 19412179 10.1038/ng.343

[2] Ivics Z, Izsvak Z, Minter A, Hackett PB (1996) Identification of functional domains and evolution of Tc1-like transposable elements. Proc Natl Acad Sci U S A 93: 5008–5013. 8643520 10.1073/pnas.93.10.5008 PMC39397

[3] Ivics Z, Hackett P, Plasterk R, Izsvak Z (1997) Molecular reconstruction of Sleeping Beauty, a Tc1-like transposon from fish, and its transposition in human cells. Cell 91: 501–510. 9390559 10.1016/s0092-8674(00)80436-5

[4] Hackett PB, Largaespada DA, Cooper LJ (2010) A transposon and transposase system for human application. Mol Ther 18: 674–683. 20104209 10.1038/mt.2010.2 PMC2862530

[5] Ivics Z, Kaufman C, Zayed H, Miskey C, Walisko O, et al (2004) The Sleeping Beauty transposable element: evolution, regulation and genetic applications. Current issues in molecular biology 6: 43–55. 14632258

[6] Cui Z, Geurts A, Liu G, Kaufman C, Hackett P (2002) Structure-function analysis of the inverted terminal repeats of the sleeping beauty transposon. Journal of molecular biology 318: 1221–1235. 12083513 10.1016/s0022-2836(02)00237-1

[7] Izsvak Z, Ivics Z, Plasterk R (2000) Sleeping Beauty, a wide host-range transposon vector for genetic transformation in vertebrates. Journal of molecular biology 302: 93–102. 10964563 10.1006/jmbi.2000.4047

[8] Zayed H, Izsvak Z, Walisko O, Ivics Z (2004) Development of hyperactive sleeping beauty transposon vectors by mutational analysis. Mol Ther 9: 292–304. 14759813 10.1016/j.ymthe.2003.11.024

[9] Ivics Z, Garrels W, Mates L, Yau TY, Bashir S, et al (2014) Germline transgenesis in pigs by cytoplasmic microinjection of Sleeping Beauty transposons. Nat Protoc 9: 810–827. 24625780 10.1038/nprot.2014.010

[10] Garrels W, Holler S, Taylor U, Herrmann D, Niemann H, et al (2014) Assessment of fetal cell chimerism in transgenic pig lines generated by sleeping beauty transposition. PLoS One 9: e96673. 24811124 10.1371/journal.pone.0096673 PMC4014516

[11] Ivics Z, Hiripi L, Hoffmann OI, Mates L, Yau TY, et al (2014) Germline transgenesis in rabbits by pronuclear microinjection of Sleeping Beauty transposons. Nat Protoc 9: 794–809. 24625779 10.1038/nprot.2014.009

[12] Katter K, Geurts AM, Hoffmann O, Mates L, Landa V, et al (2013) Transposon-mediated transgenesis, transgenic rescue, and tissue-specific gene expression in rodents and rabbits. FASEB J 27: 930–941. 23195032 10.1096/fj.12-205526 PMC3574282

[13] Ivics Z, Mates L, Yau TY, Landa V, Zidek V, et al (2014) Germline transgenesis in rodents by pronuclear microinjection of Sleeping Beauty transposons. Nat Protoc 9: 773–793. 24625778 10.1038/nprot.2014.008

[14] Jin Z, Maiti S, Huls H, Singh H, Olivares S, et al (2011) The hyperactive Sleeping Beauty transposase SB100X improves the genetic modification of T cells to express a chimeric antigen receptor. Gene Ther 18: 849–856. 21451576 10.1038/gt.2011.40 PMC4083583

[15] Liu L, Sanz S, Heggestad AD, Antharam V, Notterpek L, et al (2004) Endothelial targeting of the Sleeping Beauty transposon within lung. Mol Ther 10: 97–105. 15233946 10.1016/j.ymthe.2004.04.006

[16] Belur LR, Frandsen JL, Dupuy AJ, Ingbar DH, Largaespada DA, et al (2003) Gene insertion and long-term expression in lung mediated by the Sleeping Beauty transposon system. Mol Ther 8: 501–507. 12946324 10.1016/s1525-0016(03)00211-9

[17] Zhu J, Kren B, Park C, Bilgim R, Wong P, et al (2007) Erythroid-specific expression of beta-globin by the sleeping beauty transposon for Sickle cell disease. Biochemistry 46: 6844–6858. 17508724 10.1021/bi6024484 PMC3893920

[18] Wilber A, Linehan JL, Tian X, Woll PS, Morris JK, et al (2007) Efficient and stable transgene expression in human embryonic stem cells using transposon-mediated gene transfer. Stem Cells 25: 2919–2927. 17673526 10.1634/stemcells.2007-0026

[19] Grabundzija I, Wang J, Sebe A, Erdei Z, Kajdi R, et al (2013) Sleeping Beauty transposon-based system for cellular reprogramming and targeted gene insertion in induced pluripotent stem cells. Nucleic Acids Res 41: 1829–1847. 23275558 10.1093/nar/gks1305 PMC3561994

[20] Ammar I, Gogol-Doring A, Miskey C, Chen W, Cathomen T, et al (2012) Retargeting transposon insertions by the adeno-associated virus Rep protein. Nucleic acids research 40: 6693–6712. 22523082 10.1093/nar/gks317 PMC3413126

[21] Huang X, Guo H, Tammana S, Jung Y-C, Mellgren E, et al (2010) Gene transfer efficiency and genome-wide integration profiling of Sleeping Beauty, Tol2, and piggyBac transposons in human primary T cells. Mol Ther 18: 1803–1813. 20606646 10.1038/mt.2010.141 PMC2951558

[22] Huang X, Wilber A, Bao L, Tuong D, Tolar J, et al (2006) Stable gene transfer and expression in human primary T cells by the Sleeping Beauty transposon system. Blood 107: 483–491. 16189271 10.1182/blood-2005-05-2133 PMC1895607

[23] Voigt K, Gogol-Doring A, Miskey C, Chen W, Cathomen T, et al (2012) Retargeting sleeping beauty transposon insertions by engineered zinc finger DNA-binding domains. Mol Ther 20: 1852–1862. 22776959 10.1038/mt.2012.126 PMC3464645

[24] Borradori L, Chavanas S, Schaapveld R, Gagnoux-Palacios L, Calafat J, et al (1998) Role of the bullous pemphigoid antigen 180 (BP180) in the assembly of hemidesmosomes and cell adhesion–reexpression of BP180 in generalized atrophic benign epidermolysis bullosa keratinocytes. Experimental cell research 239: 463–476. 9521865 10.1006/excr.1997.3923

[25] McCormack W, Seiler M, Bertin T, Ubhayakar K, Palmer D, et al (2006) Helper-dependent adenoviral gene therapy mediates long-term correction of the clotting defect in the canine hemophilia A model. Journal of thrombosis and haemostasis 4: 1218–1225. 16706963 10.1111/j.1538-7836.2006.01901.x PMC3947717

[26] Sambrook J, Russell DW (2001) Molecular cloning: a laboratory manual. CSHL press 1

[27] Schmidt M, Hoffmann G, Wissler M, Lemke N, Mussig A, et al (2001) Detection and direct genomic sequencing of multiple rare unknown flanking DNA in highly complex samples. Hum Gene Ther 12: 743–749. 11339891 10.1089/104303401750148649

[28] Smit AFA, Hubley R, Green P (1996-2010) RepeatMasker Open-3.0. Available: http://repeatmasker.org. Accessed 2014 Oct 23.

[29] Cattoglio C, Facchini G, Sartori D, Antonelli A, Miccio A, et al (2007) Hot spots of retroviral integration in human CD34+ hematopoietic cells. Blood 110: 1770–1778. 17507662 10.1182/blood-2007-01-068759

[30] Cavazza A, Cocchiarella F, Bartholomae C, Schmidt M, Pincelli C, et al (2013) Self-inactivating MLV vectors have a reduced genotoxic profile in human epidermal keratinocytes. Gene Ther 20: 949–957. 23615186 10.1038/gt.2013.18

[31] Garrels W, Mates L, Holler S, Dalda A, Taylor U, et al (2011) Germline transgenic pigs by Sleeping Beauty transposition in porcine zygotes and targeted integration in the pig genome. PLoS One 6: e23573. 21897845 10.1371/journal.pone.0023573 PMC3163581

[32] Hackett P, Largaespada D, Switzer K, Cooper L (2013) Evaluating risks of insertional mutagenesis by DNA transposons in gene therapy. Translational research. 10.1016/j.trsl.2012.12.005 PMC3602164 23313630

[33] Yant S, Wu X, Huang Y, Garrison B, Burgess S, et al (2005) High-resolution genome-wide mapping of transposon integration in mammals. Molecular and cellular biology 25: 2085–2094. 15743807 10.1128/MCB.25.6.2085-2094.2005 PMC1061620

[34] Zhang W, Muck-Hausl M, Wang J, Sun C, Gebbing M, et al (2013) Integration profile and safety of an adenovirus hybrid-vector utilizing hyperactive sleeping beauty transposase for somatic integration. PLoS One 8: e75344. 24124483 10.1371/journal.pone.0075344 PMC3790794

[35] de Jong J, Akhtar W, Badhai J, Rust AG, Rad R, et al (2014) Chromatin landscapes of retroviral and transposon integration profiles. PLoS Genet 10: e1004250. 24721906 10.1371/journal.pgen.1004250 PMC3983033

[36] Wang Y, Wang J, Devaraj A, Singh M, Jimenez Orgaz A, et al (2014) Suicidal autointegration of sleeping beauty and piggyBac transposons in eukaryotic cells. PLoS Genet 10: e1004103. 24625543 10.1371/journal.pgen.1004103 PMC3952818

[37] Yant S, Ehrhardt A, Mikkelsen J, Meuse L, Pham T, et al (2002) Transposition from a gutless adeno-transposon vector stabilizes transgene expression in vivo. Nature biotechnology 20: 999–1005. 10.1038/nbt738 12244327

[38] Moldt B, Miskey C, Staunstrup N, Gogol-Doring A, Bak R, et al (2011) Comparative genomic integration profiling of Sleeping Beauty transposons mobilized with high efficacy from integrase-defective lentiviral vectors in primary human cells. Mol Ther 19: 1499–1510. 21468003 10.1038/mt.2011.47 PMC3149173

[39] Field AC, Vink C, Gabriel R, Al-Subki R, Schmidt M, et al (2013) Comparison of lentiviral and sleeping beauty mediated alphabeta T cell receptor gene transfer. PLoS One 8: e68201. 23840834 10.1371/journal.pone.0068201 PMC3695921

[40] Galla M, Schambach A, Falk C, Maetzig T, Kuehle J, et al (2011) Avoiding cytotoxicity of transposases by dose-controlled mRNA delivery. Nucleic acids research 39: 7147–7160. 21609958 10.1093/nar/gkr384 PMC3167617

[41] Zhang W, Solanki M, Muther N, Ebel M, Wang J, et al (2013) Hybrid adeno-associated viral vectors utilizing transposase-mediated somatic integration for stable transgene expression in human cells. PloS one 8 10.1371/journal.pone.0076771 PMC3792901 24116154

[42] Moiani A, Paleari Y, Sartori D, Mezzadra R, Miccio A, et al (2012) Lentiviral vector integration in the human genome induces alternative splicing and generates aberrant transcripts. The Journal of clinical investigation 122: 1653–1666. 22523069 10.1172/JCI61852 PMC3347495

[43] Cesana D, Sgualdino J, Rudilosso L, Merella S, Naldini L, et al (2012) Whole transcriptome characterization of aberrant splicing events induced by lentiviral vector integrations. The Journal of clinical investigation 122: 1667–1676. 22523064 10.1172/JCI62189 PMC3336994

[44] Titeux M, Pendaries V, Zanta-Boussif MA, Decha A, Pironon N, et al (2010) SIN retroviral vectors expressing COL7A1 under human promoters for ex vivo gene therapy of recessive dystrophic epidermolysis bullosa. Mol Ther 18: 1509–1518. 20485266 10.1038/mt.2010.91 PMC2927071

[45] Holkers M, Maggio I, Liu J, Janssen JM, Miselli F, et al (2013) Differential integrity of TALE nuclease genes following adenoviral and lentiviral vector gene transfer into human cells. Nucleic Acids Res 41: e63. 23275534 10.1093/nar/gks1446 PMC3597656

[46] Aiuti A, Cassani B, Andolfi G, Mirolo M, Biasco L, et al (2007) Multilineage hematopoietic reconstitution without clonal selection in ADA-SCID patients treated with stem cell gene therapy. The Journal of clinical investigation 117: 2233–2240. 17671653 10.1172/JCI31666 PMC1934603

[47] Biffi A, Bartolomae C, Cesana D, Cartier N, Aubourg P, et al (2011) Lentiviral vector common integration sites in preclinical models and a clinical trial reflect a benign integration bias and not oncogenic selection. Blood 117: 5332–5339. 21403130 10.1182/blood-2010-09-306761

[48] Bushman F, Lewinski M, Ciuffi A, Barr S, Leipzig J, et al (2005) Genome-wide analysis of retroviral DNA integration. Nature reviews Microbiology 3: 848–858. 16175173 10.1038/nrmicro1263

[49] Bushman F (2007) Retroviral integration and human gene therapy. The Journal of clinical investigation 117: 2083–2086. 17671645 10.1172/JCI32949 PMC1934602

[50] Montini E, Cesana D, Schmidt M, Sanvito F, Bartholomae C, et al (2009) The genotoxic potential of retroviral vectors is strongly modulated by vector design and integration site selection in a mouse model of HSC gene therapy. The Journal of clinical investigation 119: 964–975. 19307726 10.1172/JCI37630 PMC2662564

[51] Liu G, Geurts AM, Yae K, Srinivasan AR, Fahrenkrug SC, et al (2005) Target-site preferences of Sleeping Beauty transposons. J Mol Biol 346: 161–173. 15663935 10.1016/j.jmb.2004.09.086

[52] Vigdal T, Kaufman C, Izsvak Z, Voytas D, Ivics Z (2002) Common physical properties of DNA affecting target site selection of sleeping beauty and other Tc1/mariner transposable elements. Journal of molecular biology 323: 441–452. 12381300 10.1016/s0022-2836(02)00991-9

[53] Olson W, Zhurkin V (2011) Working the kinks out of nucleosomal DNA. Current opinion in structural biology 21: 348–357. 21482100 10.1016/j.sbi.2011.03.006 PMC3112303

[54] Foltz D, Jansen L, Black B, Bailey A, Yates J, et al (2006) The human CENP-A centromeric nucleosome-associated complex. Nature cell biology 8: 458–469. 16622419 10.1038/ncb1397

[55] Masumoto H, Nakano M, Ohzeki J-I (2004) The role of CENP-B and alpha-satellite DNA: de novo assembly and epigenetic maintenance of human centromeres. Chromosome research 12: 543–556. 15289662 10.1023/B:CHRO.0000036593.72788.99

[56] De Luca M, Pellegrini G, Mavilio F (2009) Gene therapy of inherited skin adhesion disorders: a critical overview. The British journal of dermatology 161: 19–24. 19466960 10.1111/j.1365-2133.2009.09243.x

[57] Carteau S, Hoffmann C, Bushman F (1998) Chromosome structure and human immunodeficiency virus type 1 cDNA integration: centromeric alphoid repeats are a disfavored target. J Virol 72: 4005–4014. 9557688 10.1128/jvi.72.5.4005-4014.1998 PMC109628

