# Correction: Genomic Analysis of *Sleeping Beauty* Transposon Integration in Human Somatic Cells

**DOI:** 10.1371/journal.pone.0228703

**Published:** 2020-01-30

**Authors:** Giandomenico Turchiano, Maria Carmela Latella, Andreas Gogol Döring, Claudia Cattoglio, Fulvio Mavilio, Zsuzsanna Izsvák, Zoltán Ivics, Alessandra Recchia

The following information is missing from the Funding statement: Dr. Zsuzsanna Izsvák was funded by European Research Council-2011-ADG-TRANSPOSOstress– 294742.
