# Supplementary material for: Genomic Analysis of Sleeping Beauty Transposon Integration in Human Somatic Cells
Source: PLoS One. 2014 Nov 12;9(11):e112712. doi: 10.1371/journal.pone.0112712 (PMC4229213; doi:10.1371/journal.pone.0112712)
Supplement: Table S1 — List of primers used for plasmid episomial amplification, LM-PCR, and site-specific amplification of the SA-genome junctions. (DOC) [file pone.0112712.s002.doc]

| **Application** | **Primer ID** | **Sequence** | **Expected amplicon** |
| --- | --- | --- | --- |
| Ampicillin sequence detection |  |  |  |
|  | Amp For | 5'-GGTTAGCTCCTTCGGTCCTG-3' | 220bp |
|  | Amp Rev | 5'-GCTATGTGGCGCGGTATTAT-3' |  |
|  |  |  |  |
|  |  |  |  |
| Transposase sequence detection |  |  |  |
|  | SB for | 5'-GCCACTCAGCAAGGAAGAAG-3' | 240bp |
|  | SB rev | 5'-GTGTTGGAAGACCCATTTGC-3' |  |
|  |  |  |  |
|  |  |  |  |
| LM-PCR | Primer Linker | 5'-GTAATACGACTCACTATAGGGC-3' | / |
|  | 2pSA1PCR | 5'-TAAGGGACTAAGTGTATGTAAAC-3' |  |
|  | TAGpSAIR | 5'-CGTATCGCCTCCCTCGCGCCATCAGCGTA  GTGTATGTAAACTTCCGACTTC-3' |  |
|  | LinkerTAG | 5'-CTATGCGCCTTGCCAGCCCGCTCAGA  GGGCTCCGCTTAAGGGAC-3' |  |
|  |  |  |  |
| Bi-directional mapping | pSAIR | 5'-GTGTATGTAAACTTCCGACTTC-3' | / |
|  | Cl 1 chrX | LM-PCR | / |
|  | Cl 1 chr9 | 5'-TCTCTTGCTTCCTGTGGCTA-3' | 204bp |
|  | Cl 5 chr3 | 5'-ACAGCCTTTCCTCATAGATA-3' | 223bp |
|  | Cl 5 chr11 | LM-PCR | / |
|  | Cl 12 chr2 | 5'-GCCATAGAGGGATACCAAAG-3' | 188bp |
|  | Cl 12 chr21 | 5'-GGGCCTTATCTTACTCTACT-3' | 397bp |
|  | Cl 15 chr14 | LM-PCR | / |
|  | Cl 15 unk | 5'-CTTGCCTGCTTGCCTGTTT-3' | 289bp |
|  | Cl 15 chr7 | 5'-CTGCTGGAATGCTCTCACTA-3' | 120bp |
|  | Cl 15 chr6 | 5'-CATCTCCCACAGGCAAGGTT-3' | 182bp |
|  | Cl 36 chr20 | 5'-AGAAACAAACAAGCCCCACT-3' | 252bp |
|  | Cl 36 chr3 | 5'-CAAACTCCTGGCTTCAACTG-3' | 103bp |
|  | Cl 38 chr18 | LM-PCR | / |
|  | Cl 38 chr20 | LM-PCR | / |
|  | Cl 38 chr22 | 5'-ACCAAGAAAAGGACACACAT-3' | 130bp |
|  | Cl 46 chr8 | LM-PCR | / |
|  | Cl 56 chr5 | 5'-AAGTGCGTTCAATTCTGTGGCT-3' | 150bp |
|  | Cl 56 chr11 | 5'-TGTCTTCTTCCTCGGCTTAC-3' | 260bp |
|  | Cl 60 chr15 | 5'-CATTGCCTGTCCTTCATTGC-3' | 270bp |
|  | Cl 34 chr3 | 5'-CAAACTCCTGGCTTCAACTG-3' | 229bp |
|  | Cl 34 chr13 | 5'-TCCTTCTGTGACTGCCTTAT-3' | 127bp |
|  | Cl 34 chr7 | 5'-GAGACGGAGGTTACAGTGAA-3' | 90bp |
|  | Cl 34 chr5 | 5'-GTGTGCCTTTGACTGGTATC-3' | 303bp |
|  | Cl 34 chr1 | 5'-CCAACTCCTGACTTCGTGAT-3' | 198bp |
|  | Cl 34 chr2 | 5'-GGAACTCCAGAACAAAGCTG-3' | 181bp |
|  | Cl 34 chr2 | 5'-TTATTCCATGCCACCCACAA-3' | 158bp |
|  | Cl 34 chr14 | 5'-GTTAGGGATTAGAAGGTAGG-3' | 90bp |
|  |  |  |  |

**Table S1.**
