# Supplementary material for: Genomic Analysis of Sleeping Beauty Transposon Integration in Human Somatic Cells
Source: PLoS One. 2014 Nov 12;9(11):e112712. doi: 10.1371/journal.pone.0112712 (PMC4229213; doi:10.1371/journal.pone.0112712)
Supplement: Table S2 — Transposed clones were analysed to show the following parameters: number of retrieved Venus+ clones for each bulk; percentage of Venus+ cells in bulk populations 48 hours p.t.; percentage of stable Venus expressing cells in bulk populations; percentage of clones positive for the Ampicillin or SB100X sequence carried by transfected plasmids; mean copy number retrieved by Southern blot analysis; recombinant events detected in transposed clones by Southern blot analysis. (DOC) [file pone.0112712.s003.doc]

**Table S2.**

| **Sample** | **# clones** | **Venus^+^ % 48h p.t.** | **Stable Venus^+^ %** | **Amp^+^ %** | **SB100X^+^ %** | **Mean Copy number** | **Recombinant events** |
| --- | --- | --- | --- | --- | --- | --- | --- |
| GABEB T2 3.2 | 29 | 64.4 | 33.3 | 21 | 0 | 4.3 | 0 |
| GABEB T2 10 | 20 | 50.3 | 20.3 | 15 | 0 | 3 | 1 |
| GABEB SA 9.7 | 34 | 42.6 | 14.9 | 14.7 | 5.5 | 1.3 | 0 |
| GABEB SA 4.8 | 25 | 20.6 | 17.4 | 20 | 8 | 3 | 0 |
| HeLa SA 9.7 | 26 | 57.5 | 23 | 11.5 | 0 | 2.4 | NA |
| HeLa SA 8.8 | 25 | 80 | 66 | 8 | 8 | 7.1 | 3 |
| HeLa T2 18 | 34 | 55 | 5 | 17.6 | 0 | 3 | NA |
| HeLa SA 18 | 18 | 50 | 4.5 | NA | NA | NA | NA |
